# Supplementary material for: Virological characteristics of the SARS-CoV-2 Omicron XBB.1.5 variant
Source: Nat Commun. 2024 Feb 8;15:1176. doi: 10.1038/s41467-024-45274-3 (PMC10853506; doi:10.1038/s41467-024-45274-3)
Supplement: Supplementary file 1 — Supplementary Information [file 41467_2024_45274_MOESM1_ESM.pdf]

Supplementary Table 1. Cryo-EM data collection, refinement and validation statistics, realated to Fig. 3

	SARS-CoV-2 XBB.1.5 spike		SARS-CoV-2 XBB.1.5 spike-ACE2		
**Data collection and processing**	**closed-1**	**closed-2**	**1RBD-up**	**2RBD-up**	**RBD-ACE2 interface**
EMDB ID	8JYK	8JYM	8JYN	8JYO	8JYP
PDB ID	36724	36726	36727	36728	36729
Microscope	Krios G4		Krios G4		
Camera	Gatan K3		Gatan K3		
energy filter	Gatan Biocontinuum		Gatan Biocontinuum		
slit width	20		20		
Magnification	130,000		130,000		
Recording mode	counting		counting		
Voltage (kV)	300		300		
Electron exposure (e–/Å^2)	56.0		56.0		
Exposure time (s)	1.5		1.5		
Number of raw frames	50		50		
Defocus range (μm)	-0.7 to -1.9		-0.7 to -1.9		
Pixel size (Å)	0.67		0.67		
Initial particle images (no.)	1,201,208		1,831,441		
Final particle images (no.)	84,161	67,040	58,221	43,957	108,121
Symmetry imposed	C3	C3	C1	C1	C1
Map resolution (Å)					
FSC 0.143	2.59	2.79	3.04	3.20	3.38
**Refinement**					
Initial model used (PDB code)	8IOS	8IOT	8IOU	8IOU, 8IOV	8IOV
Model composition					
Protein residues	3183	3099	3577	4193	795
Ligands	NAG:54	NAG:48	BMA:2, NAG:55, MAN:2	BMA:4, NAG:68, MAN:4	BMA:2, NAG:10, MAN:2
Map CC	0.86	0.85	0.86	0.86	0.84
R.m.s. deviations					
Bond lengths (Å)	0.002	0.003	0.002	0.004	0.002
Bond angles (°)	0.497	0.499	0.476	0.536	0.539
Validation					
MolProbity score	1.38	1.51	1.48	1.54	1.38
Clashscore	3.62	4.75	4.51	5.55	5.18
Rotamer outliers (%)	0	0	0	0	0
Ramachandran plot					
Favored (%)	0	0	0.03	0	0
Allowed (%)	3.53	3.83	3.72	3.6	2.53
Outliers (%)	96.47	96.17	96.25	96.4	97.47

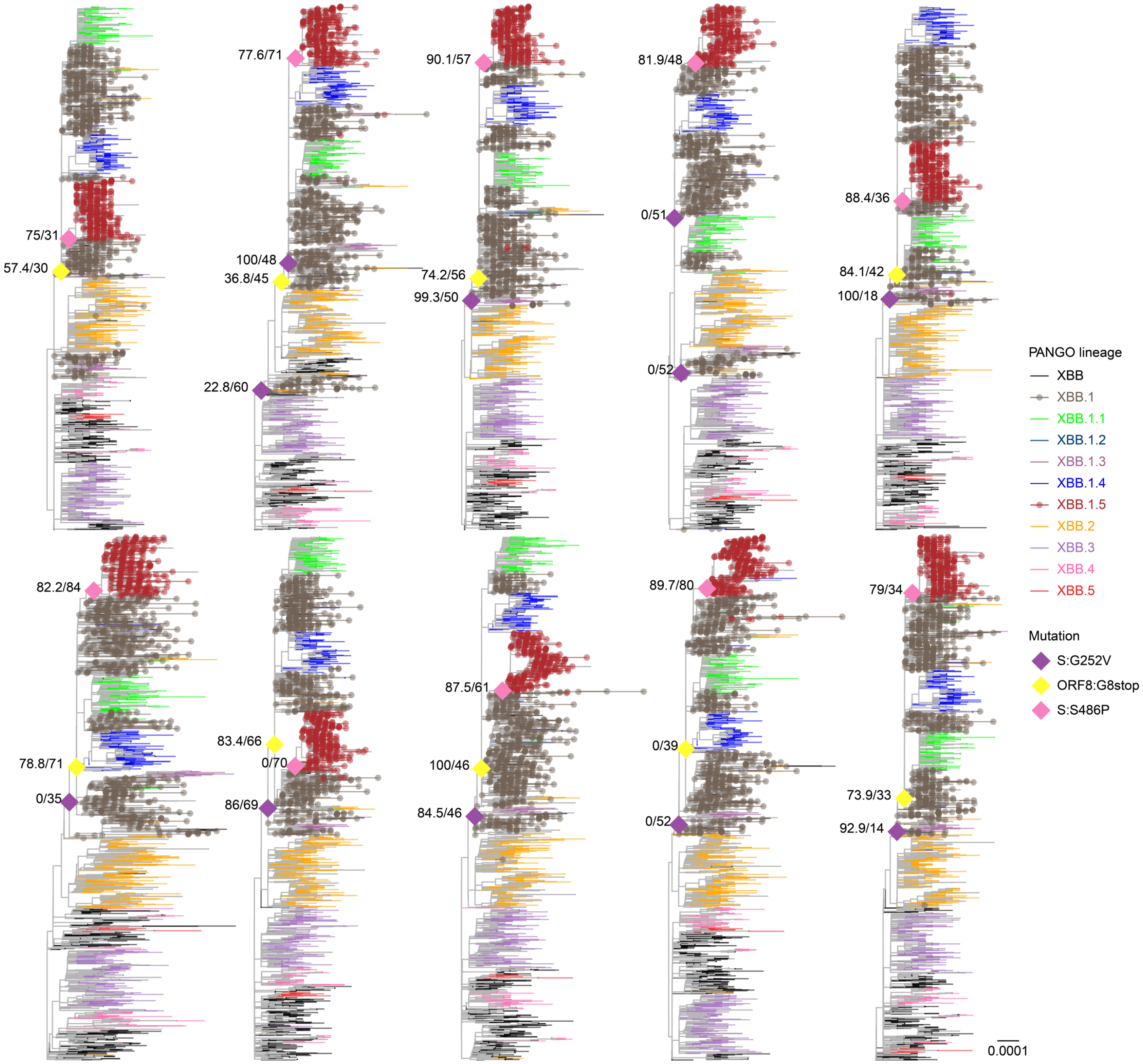

Supplementary Fig. 1. Sensitivity analysis on sequence down-sampling

The ten maximum likelihood-based phylogenetic trees of SARS-CoV-2 in the XBB lineage used in the sensitivity analysis. The bottom-right tree is the tree shown in **Fig. 1**. The XBB.1.4.1, XBB.3.1, and XBB.4.1 sublineages are included in the XBB.1.4, XBB.3, and XBB.4 lineages, respectively. Diamonds represent the occurrence of mutations of interest. Only mutation occurrences at internal nodes with at least 20 and also a half of descendant tips harboring the mutation are shown. Numbers at diamonds represent Shimodaira-Hasegawa-like approximate likelihood ratio test and ultrafast bootstrap supporting values, respectively.

Source data are provided with this paper.

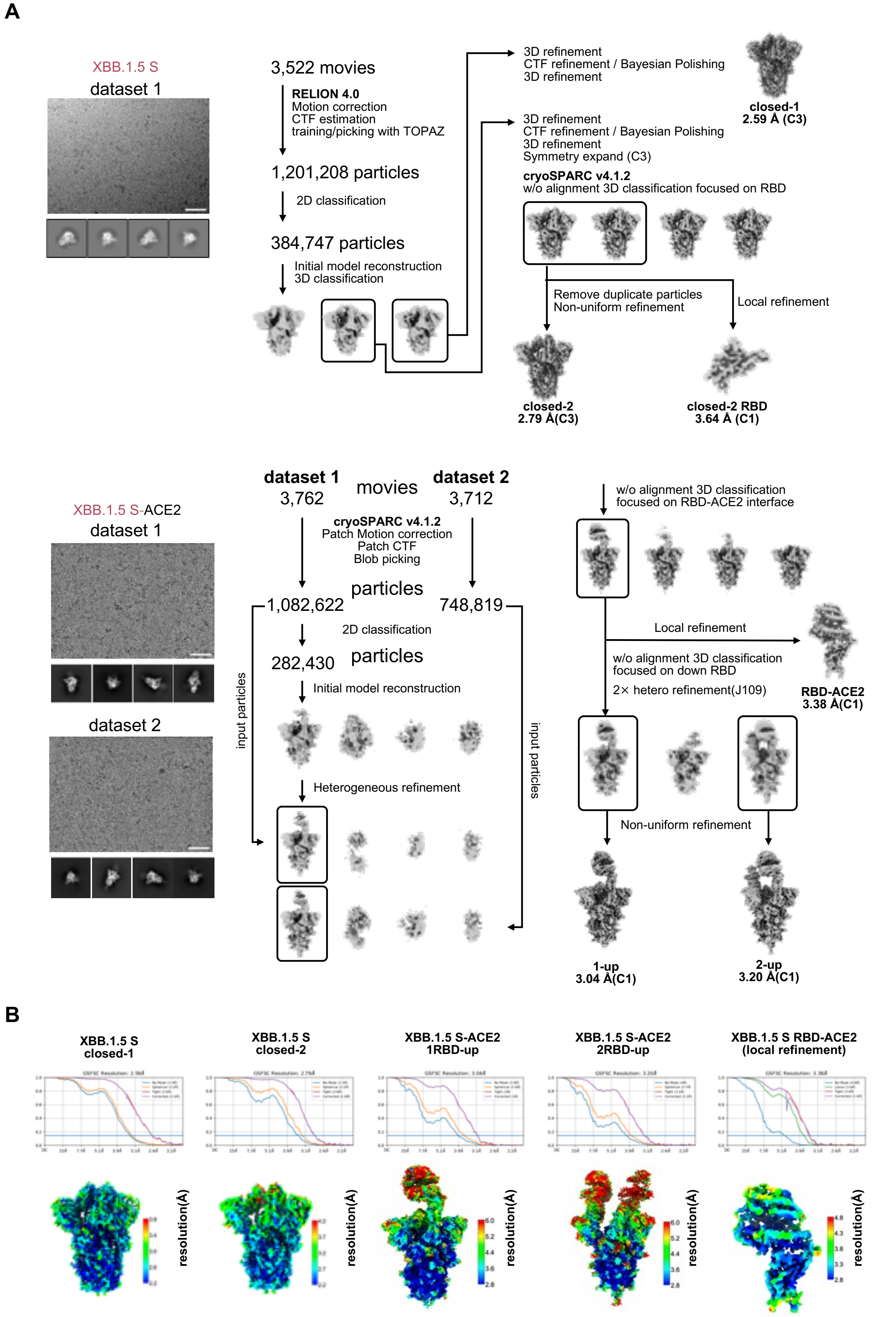

Supplementary Fig. 2. Workflow of cryo-EM data processing related to Fig. 3
(**A**) (**Left**) Representative micrograph (scale bars, 50 nm) and 2D class images. (**Right**) Cryo-EM data processing flowchart for XBB.1.5 S and XBB.1.5 S–ACE2.
(**B**) Global resolution assessment of cryo-EM maps by gold-standard Fourier shell correlation (FSC) curves at the 0.143 criteria. Local resolution is colored blue to red at each range.

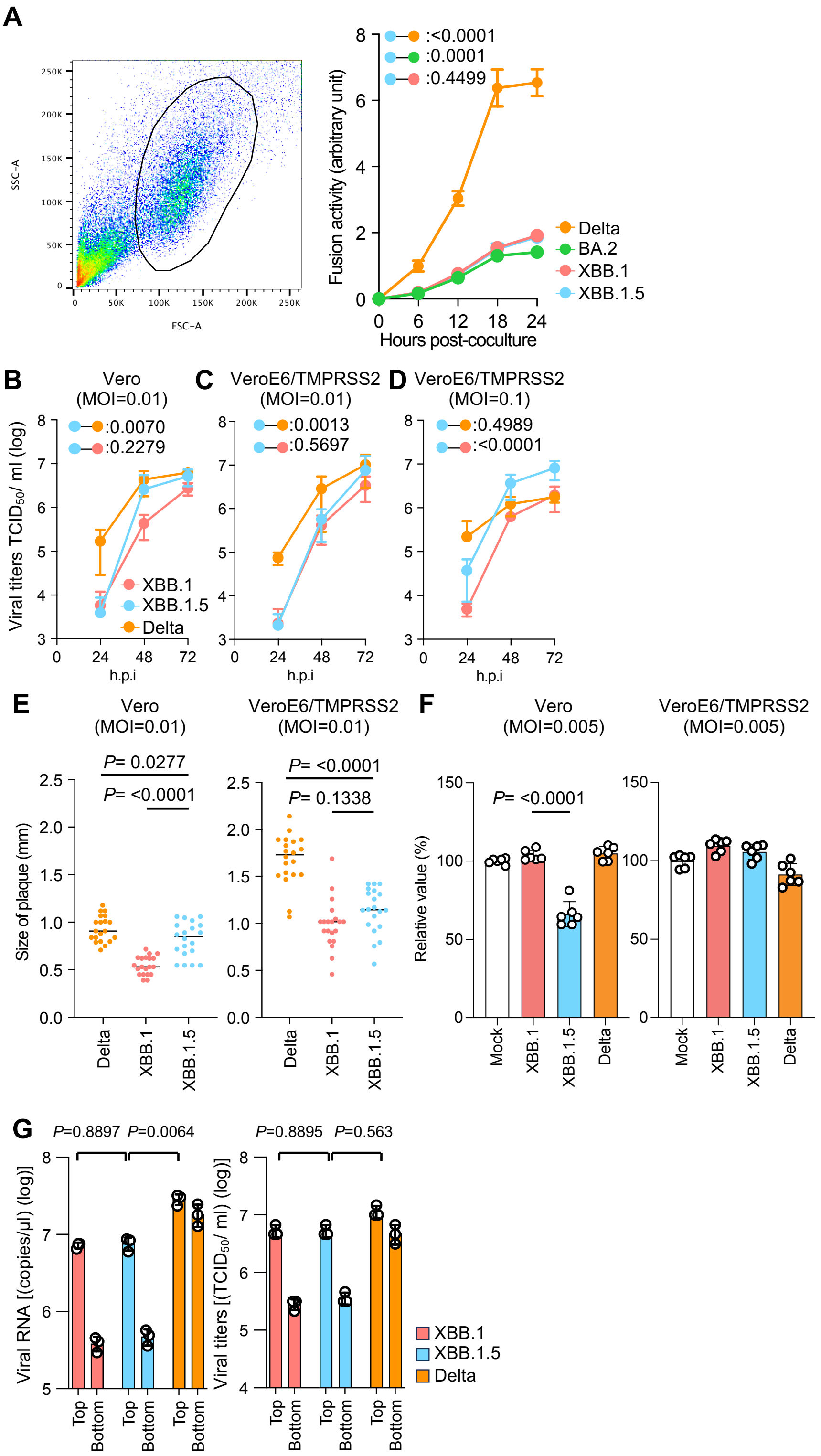

Supplementary Fig. 3. *In vitro* virological characteristics of SARS-CoV-2 variants used in this study related to Fig. 4

(**A**) (**Left**) Fusion activity (arbitrary units) of the Delta, BA.2, XBB.1, and XBB.1.5 S proteins are shown. (**Right**) A representative dot plot for the gating for flowcytometry is shown. FSC, forward scatter, SSC, side scatter. (**B**, **C**, and **D**) Infectious titers of supernatants were calculated by the $TCID_{50}$ assay and were shown in the bar graphs. (**E**) Plaque assay. Vero cells (**left**) and VeroE6/TMPRSS2 cells (**right**) were used for the target cells. A summary of the recorded plaque diameters (20 plaques per virus) is shown as bar graphs. (**F**) Viral ToxGlo assay. Clinical isolates of Delta, XBB.1, and XBB.1.5 were inoculated into Vero cells and VeroE6/TMPRSS2 cells. The cytopathic effects were quantified at 72 hpi. (**G**) Infectious titers in the top (**left**) and bottom (**middle**) channels of an airway-on-a-chip upon infection with XBB.1, XBB.1.5, and Delta are shown. Statistically significant differences versus XBB.1 across timepoints were determined by multiple regression.

Source data are provided with this paper.

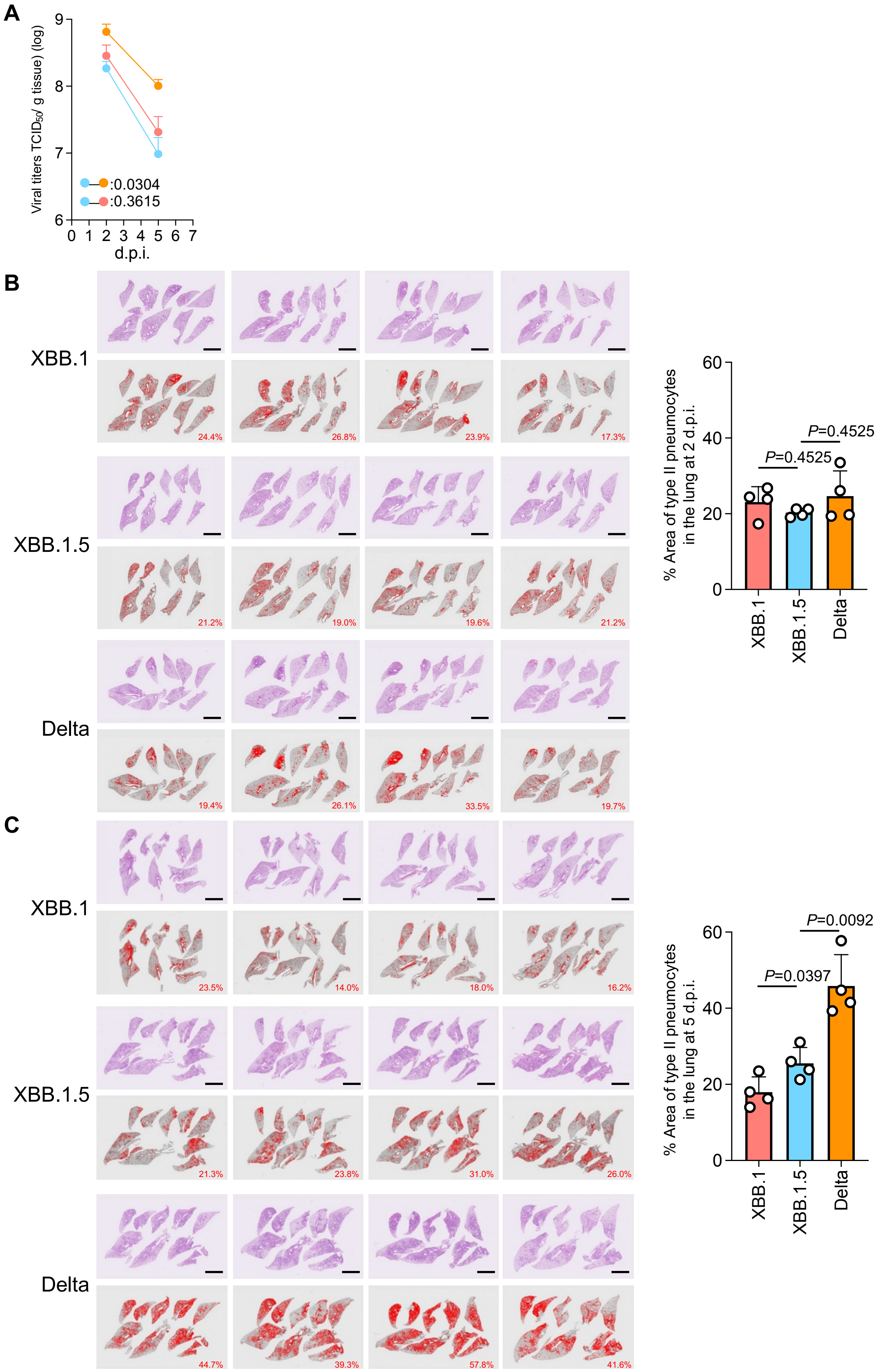

Supplementary Fig. 4. Histological observations in infected hamsters related to Fig. 5
(**A**) The viral infectious titers in the lungs were measured and calculated as $TCID_{50}$. Type II pneumocytes in the lungs of infected hamsters at (**B**) 2 d.p.i. and (**C**) 5 d.p.i (4 hamsters per infection group). H&E staining (top) and the digitalized inflammatory area with type II pneumocytes (bottom, indicated in red) are shown. The red numbers in the bottom panels indicate the percentage of the inflammatory area with type II pneumocytes. Summarized data are shown in a bar graph (**right**). Representative images are shown in **Fig. 5D**. Scale bars, 5 mm.
Source data are provided with this paper.

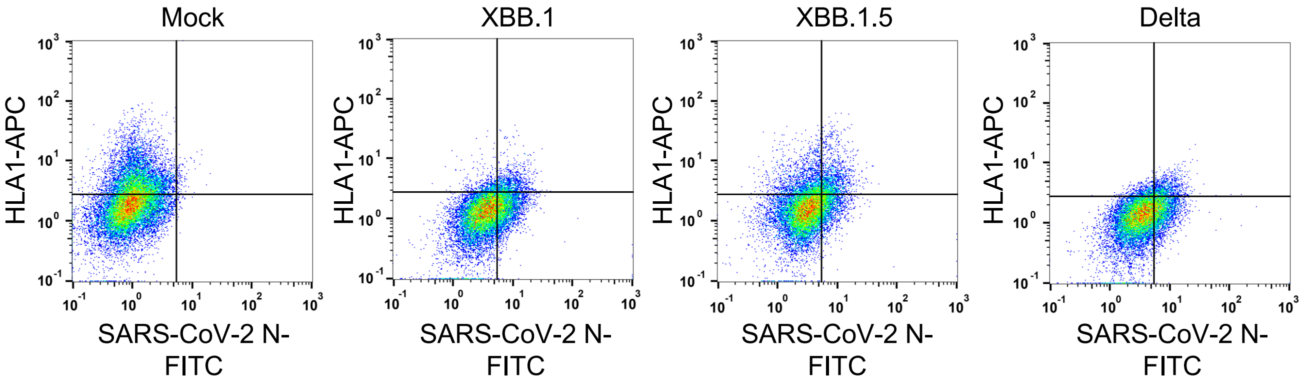

Supplementary Fig. 5. HLA-I and NP expression analysis in infected human lung organoids related to Fig. 6

Clinical isolates of XBB.1, XBB.1.5, and Delta were inoculated into human lung organoids. Two-dimensional dot plots of flow cytometry data showing the fluorescence intensity of HLA-I- and NP-positive cells.
