# Supplementary material for: Virological characteristics of the SARS-CoV-2 Omicron XBB.1.5 variant
Source: Nat Commun. 2024 Feb 8;15:1176. doi: 10.1038/s41467-024-45274-3 (PMC10853506; doi:10.1038/s41467-024-45274-3)
Supplement: Supplementary file 3 — Description of Additional Supplementary Files [file 41467_2024_45274_MOESM3_ESM.docx]

**Description of additional supplementary files**

Title: Supplementary Data 1

Description: GISAID Accession ID of SARS-CoV-2 genomic sequences used in phylogenetic tree and ancestral genomic sequence reconstruction

Title: Supplementary Data 2

Description: Human sera used in this study, related to Figure 2

Title: Supplementary Data 3

Description: Primers used in this study
