# Supplementary material for: Virological characteristics of the SARS-CoV-2 Omicron XBB.1.5 variant
Source: Nat Commun. 2024 Feb 8;15:1176. doi: 10.1038/s41467-024-45274-3 (PMC10853506; doi:10.1038/s41467-024-45274-3)
Supplement: Supplementary file 7 — Reporting Summary [file 41467_2024_45274_MOESM7_ESM.pdf]

nature portfolio

Corresponding author(s): Shinya Tanaka, Kei Sato, Takao Hashiguchi, Kazuo Takayama, Takasuke Fukuhara

Last updated by author(s): Jan 10, 2024

Reporting Summary

Nature Portfolio wishes to improve the reproducibility of the work that we publish. This form provides structure for consistency and transparency in reporting. For further information on Nature Portfolio policies, see our Editorial Policies and the Editorial Policy Checklist.

Statistics

For all statistical analyses, confirm that the following items are present in the figure legend, table legend, main text, or Methods section.

n/a	Confirmed	
[ ]	[x]	The exact sample size (*n*) for each experimental group/condition, given as a discrete number and unit of measurement
[ ]	[x]	A statement on whether measurements were taken from distinct samples or whether the same sample was measured repeatedly
[ ]	[x]	The statistical test(s) used AND whether they are one- or two-sided<br>*Only common tests should be described solely by name; describe more complex techniques in the Methods section.*
[x]	[ ]	A description of all covariates tested
[x]	[ ]	A description of any assumptions or corrections, such as tests of normality and adjustment for multiple comparisons
[ ]	[x]	A full description of the statistical parameters including central tendency (e.g. means) or other basic estimates (e.g. regression coefficient) AND variation (e.g. standard deviation) or associated estimates of uncertainty (e.g. confidence intervals)
[ ]	[x]	For null hypothesis testing, the test statistic (e.g. *F*, *t*, *r*) with confidence intervals, effect sizes, degrees of freedom and *P* value noted<br>*Give P values as exact values whenever suitable.*
[ ]	[x]	For Bayesian analysis, information on the choice of priors and Markov chain Monte Carlo settings
[x]	[ ]	For hierarchical and complex designs, identification of the appropriate level for tests and full reporting of outcomes
[x]	[ ]	Estimates of effect sizes (e.g. Cohen's *d*, Pearson's *r*), indicating how they were calculated

Our web collection on statistics for biologists contains articles on many of the points above.

Software and code

Policy information about availability of computer code

Data collection	GloMax explorer multimode microplate reader 3500 (Promega), Centro XS3 LB960 (Berthhold Technologies), MACSQuant Analyzer (Miltenyi Biotec), FACS Canto II (BD Biosciences), QuantStudio 1 Real-Time PCR system (Thermo Fisher Scientific), QuantStudio 3 Real-Time PCR system (Thermo Fisher Scientific), QuantStudio 5 Real-Time PCR system (Thermo Fisher Scientific), StepOne Plus Real-Time PCR system (Thermo Fisher Scientific), CFX Connect Real-Time PCR Detection system (Bio-Rad), Eco Real-Time PCR System (Illumina), qTOWER3 G Real-Time System (Analytik Jena), Thermal Cycler Dice Real Time System III (Takara) , 7500 Real-Time PCR System (Thermo Fisher Scientific), FinePointe Station and Review software v2.9.2.12849 (DSI), NDP.scan software v3.2.4 (Hamamatsu Photonics), Buxco Small Animal Whole Body Plethysmography system (DSI), All-in-one Fluorescence Microscope BZ-X810 (KEYENCE), SeqStudio Genetic Analyzer (Thermo Fisher Scientific)
Data analysis	FlowJo software v10.7.1 and v10.9 (BD Biosciences), Sequencher software v5.1 (Gene Codes Corporation), Excel software v16.16.8 (Microsoft), Prism 9 software v9.1.1 and v10.10 (GraphPad Software), Fiji software v2.2.0 (ImageJ), ViralMSA v1.1.24 (Moshiri et al., 2021), TrimAl v1.4.rev22 (Capella-Gutiérrez et al., 2009), IQ-TREE v2.2.0 (Minh et al., 2020), R v4.2.2 (R Core Team, 2023), EnvStats v2.7.0 (Millard, 2013), ggtree v3.6.2 (Yu, 2020), cryoSPARC v4.1.2 (Structura Biotechnology), UCSF Chimera v1.15, UCSF ChimeraX v1.4, UCSF Chimera v1.16, Coot v0.9.8.7, SAMtools v1.9, snpEff v5.0e, Pymol v2.5.0 (Schrodinger), Phenix v1.20, Molprobity v4.5.2, , Python v3.7, MAFFT v7.511<br>The computational codes used in phylogenetic analyses and ancestral sequence reconstruction are available in the GitHub repository (https://github.com/TheSatoLab/Omicron_XBB.1.5).

For manuscripts utilizing custom algorithms or software that are central to the research but not yet described in published literature, software must be made available to editors and reviewers. We strongly encourage code deposition in a community repository (e.g. GitHub). See the Nature Portfolio guidelines for submitting code & software for further information.

Data

Policy information about availability of data

All manuscripts must include a data availability statement. This statement should provide the following information, where applicable:

- Accession codes, unique identifiers, or web links for publicly available datasets
- A description of any restrictions on data availability
- For clinical datasets or third party data, please ensure that the statement adheres to our policy

SARS-CoV-2 genomic sequences and their related surveillance data are available from GISAID database (https://www.gisaid.org). The supplemental tables for the GISAID datasets (EPI_SET_231124cy) and the raw data of virus sequences analyzed in this study are deposited in the GitHub repository (https://github.com/TheSatoLab/Omicron_XBB.1.5). The accession numbers of viral sequences used in this study are listed in Method section. The atomic coordinates and cryo-EM maps for the structures are deposited in the PBD (https://www.rcsb.org/search) and the EMDB (https://www.ebi.ac.uk/emdb/). The atomic coordinates and cryo-EM maps for the structures of the XBB.1.5 S protein closed-1 state (PDB code: 8JYK, EMDB code: 36724), closed-2 state (PDB code: 8JYM, EMDB code: 36726), in complex with hACE2 RBD one-up state (PDB code: 8JYN, EMDB code: 36727), in complex with hACE2 RBD two-up state (PDB code: 8JYO, EMDB code: 36728), and XBB.1.5 S RBD–hACE2 (PDB code: 8JYP, EMDB code: 36729) have been deposited in the Protein Data Bank (www.rcsb.org) and Electron Microscopy Data Bank (www.ebi.ac.uk/emdb/).

Research involving human participants, their data, or biological material

Policy information about studies with human participants or human data. See also policy information about sex, gender (identity/presentation), and sexual orientation and race, ethnicity and racism.

Reporting on sex and gender	Fourth-dose vaccine sera from individuals who had been vaccinated with monovalent vaccine (15 donors; average age: 42 years, range: 30–56 years, 40% male) (Fig. 2A), BA.1 bivalent vaccine sera (20 donors; average age: 55 years, range: 30–80 years, 35% male) (Fig. 2B), and BA.5 bivalent vaccine sera (21 donors; average age: 51 years, range: 18–86 years, 48% male) (Fig. 2C) were collected.
Reporting on race, ethnicity, or other socially relevant groupings	All of the sera obtained from Japanese
Population characteristics	Fourth-dose vaccine sera from individuals who had been vaccinated with monovalent vaccine (15 donors; average age: 42 years, range: 30–56 years, 40% male) (Fig. 2A), BA.1 bivalent vaccine sera (20 donors; average age: 55 years, range: 30–80 years, 35% male) (Fig. 2B), and BA.5 bivalent vaccine sera (21 donors; average age: 51 years, range: 18–86 years, 48% male) (Fig. 2C) were collected.
Recruitment	All protocols involving specimens from human subjects recruited at Interpark Kuramochi Clinic was reviewed and approved by the Institutional Review Board of Interpark Kuramochi Clinic (approval ID: G2021-004). No potential self-selection bias or other biases in the recruitment.
Ethics oversight	All protocols involving specimens from human subjects recruited at Interpark Kuramochi Clinic were reviewed and approved by the Institutional Review Board of Interpark Kuramochi Clinic (approval ID: G2021-004). All human subjects provided written informed consent. All protocols for the use of human specimens were reviewed and approved by the Institutional Review Boards of The Institute of Medical Science, The University of Tokyo (approval IDs: 2021-1-0416 and 2021-18-0617), Kumamoto University (approval ID: 2066), and University of Miyazaki (approval ID: O-1021).

Note that full information on the approval of the study protocol must also be provided in the manuscript.

Field-specific reporting

Please select the one below that is the best fit for your research. If you are not sure, read the appropriate sections before making your selection.

[x] Life sciences [ ] Behavioural & social sciences [ ] Ecological, evolutionary & environmental sciences

For a reference copy of the document with all sections, see nature.com/documents/nr-reporting-summary-flat.pdf

Life sciences study design

All studies must disclose on these points even when the disclosure is negative.

Sample size	The sample sizes ($n > 3$) for cell culture experiments were chosen for applying statistical tests. The sample sizes ($n > 3$) for the hamster studies were chosen because they have previously been shown to be sufficient to evaluate a significant difference among groups (Belser et al., Nature, 2013; Zhang et al., Science, 2013; Imai et al., Nature Microbiology, 2020; Saito et al., Nature, 2021).
Data exclusions	No data were excluded from the analyses.
Replication	In vitro experiments representative of at least 2 experiments with multiple samples per time point. In vivo experiments (hamster) utilized multiple animals per group per time point and were from more than single experiment, independently. In vivo experiments were replicated and performed independently. All attempts at replication were successful.

Randomization	No method of randomization was used to determine how the animals were allocated to the experimental groups and processed in this study, because covariates (sex and age) were identical (male, 4 weeks old). For experiments other than animal studies, randomization is not applicable because homogenous materials (i.e., cell lines) were used. Therefore, randomization is not applicable.
Blinding	No blinding was carried out, because these are not relevant for an observational study.

Reporting for specific materials, systems and methods

We require information from authors about some types of materials, experimental systems and methods used in many studies. Here, indicate whether each material, system or method listed is relevant to your study. If you are not sure if a list item applies to your research, read the appropriate section before selecting a response.

Materials & experimental systems

n/a	Involved in the study
☐	☒ Antibodies
☐	☒ Eukaryotic cell lines
☒	☐ Palaeontology and archaeology
☐	☒ Animals and other organisms
☒	☐ Clinical data
☒	☐ Dual use research of concern
☒	☐ Plants

Methods

n/a	Involved in the study
☒	☐ ChIP-seq
☐	☒ Flow cytometry
☒	☐ MRI-based neuroimaging

Antibodies

Antibodies used	For IHC:<br>mouse anti-SARS-CoV-2 N monoclonal antibody (R&D systems, Clone 1035111, Cat# MAB10474-SP, 1:400)<br>For flow cytometry:<br>rabbit anti-SARS-CoV-2 S S1/S2 polyclonal antibody (Thermo Fisher Scientific, Cat# PA5-112048, 1:100)<br>Normal rabbit IgG (Southern Biotech, Cat# 0111-01, 1:100)<br>APC-conjugated goat anti-rabbit IgG polyclonal antibody (Jackson ImmunoResearch, Cat# 111-136-144, 1:50)<br>APC-labeled anti-human HLA class 1 (R&D systems, Cat# FAB7098A)<br>anti-SARS-CoV-2 NP antibodies (BIO Vision, Cat# A2061-50)<br>anti-Rabbit IgG (H+L) cross-adsorbed secondary antibody, Alexa Fluor 488 (Thermo Fisher Scientific, Cat# A-11008).
Validation	Validation was conducted by manufacturers prior to sale, and validation statements are available on the manufacturers' website.

Eukaryotic cell lines

Policy information about cell lines and Sex and Gender in Research

Cell line source(s)	HEK293T cells (a human embryonic kidney cell line; ATCC, CRL-3216)<br>HEK293 cells (a human embryonic kidney cell line; ATCC CRL-1573)<br>HEK293S GnTI(-) cells (Reeves PJ et al., PNAS 2002)<br>HOS-ACE2/TMPRSS2 cells (HOS cells stably expressing human ACE2 and TMPRSS2) (Ozono et al., Nat Commun, 2021; Ferreira et al., J Infect Dis, 2021)<br>HEK293-ACE2 cells (HEK293 cells stably expressing human ACE2) (Motozono et al., Cell Host & Microbe, 2021)<br>HEK293-ACE2/TMPRSS2 cells (Motozono et al., Cell Host & Microbe, 2021)<br>Vero cells [an African green monkey (Chlorocebus sabaeus) kidney cell line; JCRB0111]<br>VeroE6/TMPRSS2 cells (JCRB1819) (Matsuyama et al., Proc Natl Acad Sci, 2020)<br>Calu-3 cells (a human lung epithelial cell line; ATCC HTB-55)<br>Calu-3/DSP1-7 cells (Yamamoto et al., Viruses, 2020)
Authentication	None of the cells used were authenticated.
Mycoplasma contamination	All cell lines were regularly tested for mycoplasma contamination by using PCR and were confirmed to be mycoplasma-free.
Commonly misidentified lines<br>(See ICLAC register)	No commonly misidentified cell lines were used.

Animals and other research organisms

Policy information about studies involving animals; ARRIVE guidelines recommended for reporting animal research, and Sex and Gender in Research

Laboratory animals	Syrian hamsters (male, 4 weeks old) were purchased from Japan SLC Inc. (Shizuoka, Japan).
Wild animals	No wild animals used in this study.
Reporting on sex	Epidemiological studies of the COVID-19 patients have suggested the male bias in outcomes of lung illness. In addition, hamster model, male hamsters have been reported to be more susceptible to SARS-CoV-2 infection (Lunzhi Yuan et al Signal Transduction and Targeted study, 2021). Therefore, also in this study, male hamsters were used.
Field-collected samples	No field collected sample was used in the study.
Ethics oversight	All experiments with hamsters were performed in accordance with the Science Council of Japan's Guidelines for Proper Conduct of Animal Experiments. The protocols were approved by the Institutional Animal Care and Use Committee of National University Corporation Hokkaido University (approval numbers 20-0123 and 20-0060).

Note that full information on the approval of the study protocol must also be provided in the manuscript.

Flow Cytometry

Plots

Confirm that:

- [x] The axis labels state the marker and fluorochrome used (e.g. CD4-FITC).
- [x] The axis scales are clearly visible. Include numbers along axes only for bottom left plot of group (a 'group' is an analysis of identical markers).
- [x] All plots are contour plots with outliers or pseudocolor plots.
- [x] A numerical value for number of cells or percentage (with statistics) is provided.

Methodology

Sample preparation	Clinical isolates of XBB.1, XBB.1.5, and Delta were inoculated into human iPSC-derived lung organoids. Single-cell suspensions of the infected human iPSC-derived lung organoids were treated with 1 × Permeabilization Buffer (e-Bioscience, Thermo Fisher Scientific, Cat# 00-8333-56) for FACS analysis. For fusion assay, HEK293 cells were cotransfected with S expression plasmids (400 ng) and pDSP8-11 (400 ng) using TransIT-LT1 (Takara, Cat# MIR2300).
Instrument	MACSQuant Analyzer 10 (Miltenyi Biotec), FACS Canto II (BD Biosciences)
Software	FlowJo software v10.7.1 (BD Biosciences), FlowJo software v10.9 (BD Biosciences)
Cell population abundance	10,000 or 50,000 cells gated in the FSC-A/SSC-A plot were acquired for each condition.
Gating strategy	10,000 or 50,000 cells gated in the FSC-A/SSC-A plot were acquired for each condition. For measurement of the expression level of the N protein and human HLA class 1, isotype control was used as a negative control to exclude FITC- or APC-negative population. For measurement of the surface expression level of the S protein, isotype control (normal rabbit IgG) was used as a negative control to exclude APC-negative population.

- [x] Tick this box to confirm that a figure exemplifying the gating strategy is provided in the Supplementary Information.
